# On the Preparation of the Mild Sublimate Mercury

**Published:** 1803-01-01

**Authors:** 


### *On the Preparation of the mild Sublimate Mercury, by* Mr. Buchholz; *communicated by* Dr. Noehden.

It is known that the common prescription for preparing the sweet mercury is to combine three parts of pure quicksilver with four parts of corrosive muriated quicksilver; but the Pharmacopœa Borussica differs from this prescription, by ordering five parts of corrosive sublimate mercury to be mixed with four parts of metallic quicksilver. As there remains however a part of the quicksilver, on following the common prescription, which prevents us from obtaining the mild sublimate in proper purity, Mr. Buchholz supposes this to take place in a greater degree by proceeding according to the prescription of the Pharmacopœa Borussica. In order to be more convinced of this circumstance, he accurately mixed 4lb. of corrosive sublimate with 3 lb. 3 oz. 4 sc. of metallic quicksilver, and sublimed the whole mass by a strong fire. After the sublimation, he found the greatest part of the sublimate to be impregnated with metalline quicksilver of a greyish colour. The same result ensued after a second sublimation by a still stronger fire, as the upper part of the phial was entirely covered with metalline quicksilver, the quantity of which amounted to two ounces, two drachms. When the sublimation was made in a retort, the same result appeared. All this induces Mr. B. to think the old proportion in combining the corrosive sublimate mercury with metalline quicksilver preferable to that which is recommended in the Pharmacopœa Borussica. Mr. B. farther observed, that, if the sublimation is accidentally prevented from being finished, he notwithstanding found that part, which remained unsublimed, equally changed into sweet sublimate, as that which had been really sublimed; whence he concludes, that in preparing the mild muriated mercury by a double sublimation, it might not be necessary to finish entirely the first sublimation. It is known, that Mr. Scheele recommended the preparation of sweet mercury by precipitation as an easy and expeditious method; but though this precipitate appeared, from several tests, not to differ from that prepared by sublimation, yet Mr. Buchholz thought it not improper to make several experiments, in order to ascertain whether both preparations are really different in their component particles and medical effects.

From the results of these experiments, it appears that the preparation of Scheele contains no traces of corrosive mercury; that it agrees in the essential properties with the sweet mercury, which is prepared in the common way; by the same difficulty of dissolving; by its being not precipitated with lime water as red oxyd of mercury; and it seems merely to differ by evolving a very inconsiderable portion of muriatic acid when it is sublimed. But in its medicinal properties, it is perfectly equal to the former. The preparation is less expensive and troublesome, and has different other advantages, particularly if we proceed according to the following improved method of Scheele.

Take of quicksilver and of pure dilute nitrous acid equal parts; put the mixture first in a cool place; afterwards, when the solution of the quicksilver does no longer go on, put it in a warm place; and at last, for saturating the solution entirely with quicksilver, into the sand bath, gradually increasing the heat till the solution begins to boil. In this manner we prevent any excessive combination of oxygen with the quicksilver, though we obtain a saturated solution, particularly by adding so much quicksilver that there remains a part undissolved. The solution being perfectly saturated, must be poured boiling hot into a hot solution containing as much common salt as there was employed of quicksilver for the solution. Having finished the decomposition of the nitrat of mercury by continually stirring that mixture for some minutes, and separated the saline particles by repeated edulcorations with pure water, take to every pound of quicksilver employed in the process, *one* ounce of pure sal ammoniac dissolved in *two* pints of water, which solution must be mixed with the precipitate obtained in the above way, and placed for three or four hours in the sand bath, till it boils gently. When the precipitate has subsided, pour off the solution and add it to the liquors still containing quicksilver, which have been separated from the above precipitate, after the quicksilver had been first precipitated by the common salt. This mixture being carefully decomposed by a solution of carbonat of kali, yields all the remaining quicksilver in form of a beautiful white precipitate. Wash both precipitates by frequent additions of water and subsequent decantations, until no saline taste is perceptible; dry and keep them in a shady place.

